# Which is the best method of sterilization of tumour bone for reimplantation? a biomechanical and histopathological study

**DOI:** 10.1186/1475-925X-9-48

**Published:** 2010-09-10

**Authors:** Vivek Ajit Singh, Janarthan Nagalingam, Marniza Saad, Jayalakshmi Pailoor

**Affiliations:** 1 Department of Orthopaedic Surgery, University Malaya Medical Center, 50603 Kuala Lumpur, Malaysia; 2 Department of Oncology, University Malaya Medical Center, 50603 Kuala Lumpur, Malaysia; 3 Department of Pathology, University Malaya Medical Center, 50603 Kuala Lumpur, Malaysia

## Abstract

**Introduction:**

Sterilization and re-usage of tumour bone for reconstruction after tumour resection is now gaining popularity in the East. This recycle tumour bone needs to be sterilized in order to eradicate the tumour cells before re-implantation for limb salvage procedures. The effect of some of these treatments on the integrity and sterility of the bone after treatment has been published but there has yet been a direct comparison between the various methods of sterilization to determine the one method that gives the best tumour kill without compromising the bone's structural integrity.

**Method:**

This study was performed to evaluate the effect of several sterilization methods on the mechanical behavior of human cortical bone graft and histopathology evaluation of tumour bone samples after being processed with 4 different methods of sterilization. Fresh human cortical tumour bone is harvested from the diaphyseal region of the tumour bone were sterilized by autoclave (n =10); boiling (n =10); pasteurization (n =10); and irradiation (n =10). There were also 10 control specimens that did not receive any form of sterilization treatment. The biomechanical test conducted were stress to failure, modulus and strain to failure, which were determined from axial compression testing. Statistical analysis (ANOVA) was performed on these results. Significance level (α) and power (β) were set to 0.05 and 0.90, respectively.

**Results:**

ANOVA analysis of 'failure stress', 'modulus' and 'strain to failure' demonstrated significant differences (p < 0.05) between treated cortical bone and untreated specimens under mechanical loading.

'Stress to failure' was significantly reduced in boiled, autoclaved and irradiated cortical bone samples (p < 0.05). 'Modulus' detected significant differences in the boiled, autoclaved and pasteurization specimens compared to controls (p < 0.05). 'Strain to failure' was reduced by irradiation (p < 0.05) but not by the other three methods of treatments.

Histopathology study revealed no viable tumour cell in any of four types of treatment group compared to the untreated control group.

**Conclusions:**

Sterilization of cortical bone sample by pasteurization and to a lesser extent, irradiation does not significantly alter the mechanical properties when compared with untreated samples. Mechanical properties degrade with the use of high temperature for sterilization (boiling). All methods of sterilization gave rise to 100 percent tumour kill.

## Introduction

Osteosarcoma is the most common malignant primary bone tumour. It's a high-grade tumour derived from the mesenchymal tissue. Approximately 5 per million cases per year are diagnosed in the United States [1]. Osteogenic sarcoma is the most common bone sarcoma and the third most common malignancy in children and adolescents. The most frequent sites of origin is the metaphyseal regions of the distal femur, proximal tibia and proximal humerus, although this tumour can practically develop in any bone [2,3].

The treatment of Osteosarcoma consists of neo-adjuvant chemotherapy, followed by surgery and finally supplemented by adjuvant chemotherapy. The surgery can either be an amputation or limb salvage surgery. Limb salvage surgery is a type of surgery primarily performed to adequately excise tumour while preserving the particular limb. It consists of complete removal of a malignant tumour with wide margins and reconstruction of the limb with an acceptable oncologic, functional, and cosmetic result.

In limb salvage surgery for bone sarcomas, there is usually a large bone surgical defect. As most of the bone sarcomas occur in the metaphyseal portion of the bone, the resection usually involves the whole proximal or distal portion of the bone including the joint. For tumours that involve the diaphyseal portion of a bone, an intercalary resection and reconstruction can be performed that saves the joints on either side.

However, as more radical resections have been developed, the need for suitable substitutes for the resected segment has become evident. The choice of reconstruction is dependent on several factors, which include the extent of tumour, the remaining bone and soft tissue, and the patient's physical demands and expectations.

The various methods of reconstruction available are the following:

1. Endoprosthesis replacement.

2. Allograft replacement.

3. Alloprosthetic composite.

4. Distraction osteogenesis.

5. Rotationplasty.

6. Arthrodesis.

7. Autograft.

Taking in consideration the long-term viability of the reconstruction in limb salvage surgery, the use of bone appears more appealing due the potential of bone remodeling and its incorporation with host bone. Recently there has been a great interest in recycling the tumour bone itself by various methods of sterilization and reimplantation. The methods describe in the literature are boiling, autoclaving, irradiation, immerse in alcohol, pasteurization and the use of liquid nitrogen. We carried out a direct comparison between the four commonly used methods of sterilization to determine the one that gives you the best tumour kill without compromising on the structural integrity of the bone.

### Materials and methods

This is a prospective in-vitro study. Samples were collected from patients diagnosed with Osteosarcoma during the limb salvage procedure. We collected samples from 10 consecutive operated cases starting from January 2009 till March 2009. All these patients underwent wide resection with endoprosthetic replacement.

### Specimen collection

**Figure 1:**
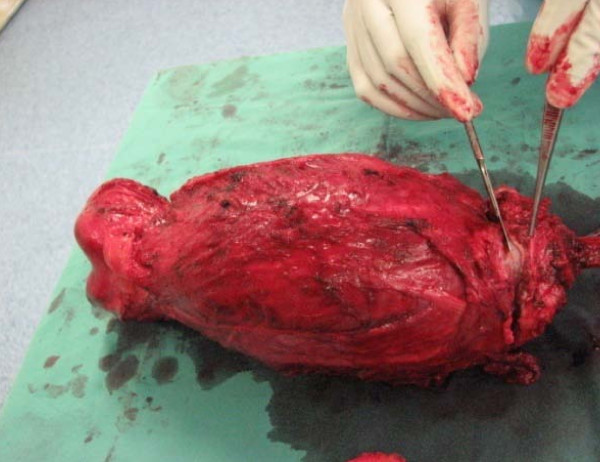
**Soft tissue and periosteum is stripped off the primary tumour bone**.

The specimens for the study were obtained from patient with Osteosarcoma involving the long bone such as distal femur or proximal tibia. The tumour bone is removed en bloc with 3 cm of normal bone margin. Resection length is based on the initial MRI (Magnetic Resonance Scanning) scan. After resection, the tumour is placed on a separate sterile table. The overlying soft tissues and periosteum is dissected out from the bone specimen. (Figure 1).

**Figure 2:**
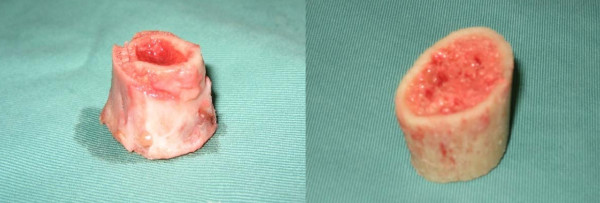
**Specimen for analysis**. The right is for biomechanical testing and left for histopathological examination.

The specimen needed for biomechanical study is measured 2 centimeters (cm) in length with a steel caliper and marked. The sample is taken from the diaphyseal region. A tubular bone sample of 2 centimeters in length is cut out (as shown in figure 2). The second specimen measuring 1 centimeter in length is cut from the tumour bone and divided into five pieces for histopathological study after undergoing sterilization.

### Specimen for biomechanical test

Bone specimens taken from the diaphyseal region is then measured and marked using a caliper. The specimen is then cut accurately to 5 pieces each measuring 2 cm × 1 cm × 0.5 cm using an electrical saw. The cutting was carried out with water cooling to prevent any rise of temperature which may lead to irreversible thermal necrosis.

**Figure 3:**
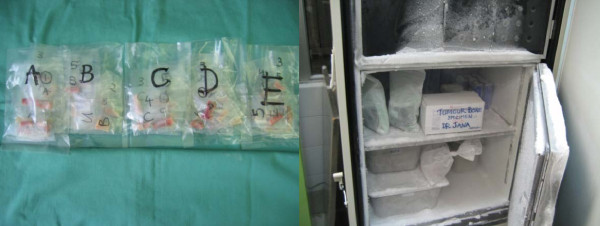
**Specimens packed separately, labeled and put in -80 degrees freezer**.

These samples are wrapped with saline gauze and placed in plastic container. Each container is labeled according to the patients name's details. A total of ten specimens were harvested from ten patients and each of these specimens was then cut into 5 equal size samples; each placed in different containers and labeled. The specimens are stored in bone fridge at -80 degree Celsius (for maximum of two weeks before treatment). All samples were kept wet, placed in heat-sealed polyethylene envelopes and stored. The 5 equal size cut samples obtained from one bone specimen are then labeled A, B, C, D and E (as shown in figure 3) For example patient 1 has samples 1A, 1B, 1C, 1 D and 1E.

Group A will undergo autoclaving, group B boiling, Group C is the control group, group D will be treated by pasteurization and group E irradiation.

### Specimens for histopathology study

**Figure 4:**
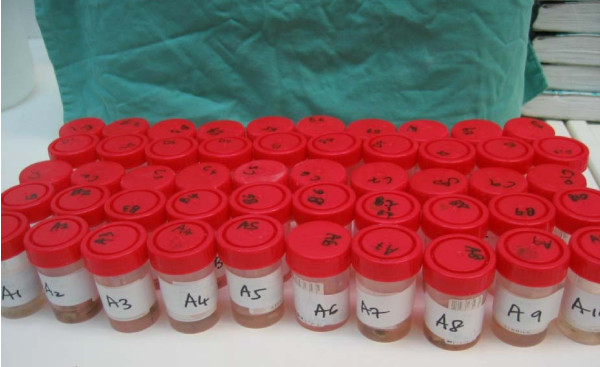
**Histopathological samples in formalin filled containers**.

Specimens containing the tumour bone collected from the resected bone are then placed in bottle containing formalin. Each bottle is labeled according to patient's name and the sterilization method (as shown in figure 4).

### Sterilization methods

Each sample is subjected to a method of sterilization accordingly:

a. Sample A was Autoclaved.

b. Sample B was boiled.

c. Sample C was used as control.

d. Sample D was pasteurized.

e. Sample E was irradiated.

### Autoclave (Sample A)

The samples are placed in an autoclave compatible package. The bone specimen is autoclaved at 135 degrees Celsius for 12 to 15 minutes at a pressure of 0.2 mega Pascal (twenty nine pounds per square inch) [4,5].

### Boiling (Sample B)

Specimens are placed in sterile bottle after de-freezing and boiled at 100 deg C for 30 minutes in normal saline.

### Control (Sample C)

These samples are not treated with any type of sterilization technique. The specimens are soaked in a saline dish for 20 minutes served as controls.

### Pasteurization (Sample D)

Specimens placed in a sterile bottle after de-freezing and kept in preheated saline at 65 C for 30 minutes (homeothermal heater).

### Irradiation (Sample E)

**Figure 5:**
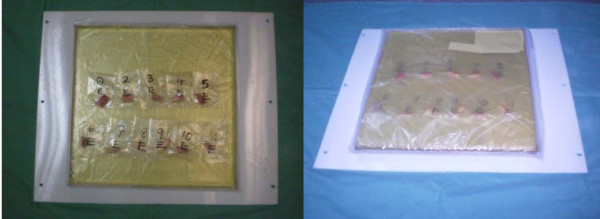
**Cortical bone specimens placed in a board cover with a silicon pad layer before Irradiation**.

Samples involved were defrosted and Irradiated with 6 MV photons from a linear accelerator. All segments were irradiated up to a total dose of 50 Gy given in single fraction (as shown in figure 5).

### Biomechanical

#### Testing Compression test

**Figure 6:**
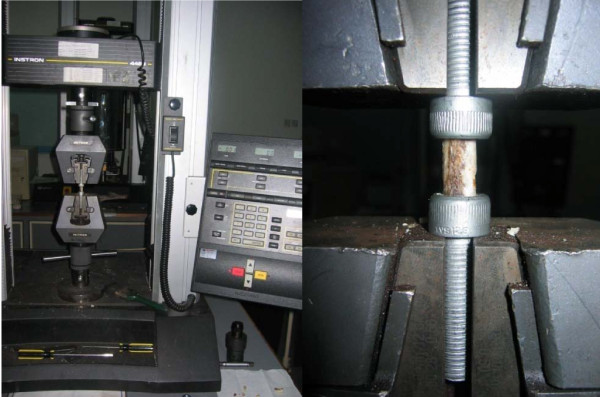
**Instron machine with sample mounted on the jig**.

The 20-mm bone graft was placed between 2 parallel stainless steel platen so that the long axis of the bone matched the compression axis using an Instron type 1026 mechanical testing machine (Instron Ltd, High Wycombe, UK). The compressive strength of the bone was measured with a speed of compression of 0.2 mm/s (as shown in figure 6). The equipment was connected to a computer in order to determine the load deformation curve, and the maximum load was measured. For each sample, the test was interrupted once the first deflection of the stress/strain curve was obtained. Deformation at the time of failure was measured, and elasticity modulus was calculated in the first, straight section of the strain deformation curve. The same strength test was performed for all 5 groups, and the data were analyzed using one-way ANOVA, and post-hoc comparisons of means. Values of *p *< 0.05 were considered statistically significant. Data are presented as means with their standard deviations. Stress direction on the studied samples, being orientated in accordance with its initial physiological situation, was consequently identical for all the samples tested. SPSS version 14 was used to analyze the data.

### Histopathology study

All specimens will be fixed with buffered formalin for histological analysis. The histological features studied include viable tumour remaining after sterilization by each of the above methods.

Fixation was carried out as soon as possible after removal of the tissues (in the case of surgical pathology).

The standard solution is 10% neutral buffered formalin. Formalin is used for all routine surgical pathology tissues when an H and E slide is to be produced.

Ethical committee approval was given for this study from the University Hospital Ethical committee.

## Results

The patients were aged between 16 to 35 years with an average age of 25 years. There were six were male and four female patients. Three patients had osteosarcoma of the distal femur and seven had osteosarcoma of the proximal tibia. All cases where in stage 2b, based on Enneking staging and all of them had been treated with adjuvant chemotherapy before the surgery. Other tumors were not included in this study.

### Mechanical testing

A one-way ANOVA revealed significant differences in the treated specimens on the compression test: 'stress to failure', 'strain to failure' and 'modulus' [p < (0.05)].

The results of 'stress to failure', 'modulus' and 'strain to failure' is shown in table 1.

**Table 1:** The results of stress to failure, modulus and strain at failure for control and treated specimens are shown.

		N	Mean	Std. Deviation	Std. Error	Minimum	Maximum
**Stress to failure**	Autoclave	10	.916	.300	.094	.58	1.63
(MPa)	Boiled	10	.882	.245	.077	.63	1.47
	Pasteurization	10	1.222	.743	.235	.46	2.59
	Irradiation	10	1.022	.617	.195	.44	2.56
	Control	10	2.035	1.253	.396	.77	4.64
	Total	50	1.215	.820	.116	.44	4.64
							
**Strain to failure**	Autoclave	10	4.671	1.926	.609	2.42	9.43
%	Boiled	10	4.699	2.716	.859	1.27	9.63
	Pasteurization	10	5.832	3.917	1.238	1.94	14.62
	Irradiation	10	3.654	1.272	.402	1.39	5.79
	Control	10	9.691	7.526	2.380	.71	25.35
	Total	50	5.709	4.481	.633	.71	25.35
							
**Modulus**	autoclave	10	6.462	4.923	1.556	1.37	13.36
(MPa)	Boiled	10	3.909	2.421	.765	1.27	9.16
	pasteur	10	7.764	5.739	1.814	2.80	18.67
	gamma	10	10.593	5.025	1.589	1.29	17.02
	Control	10	19.039	16.358	5.173	7.18	49.35
	Total	50	9.553	9.646	1.364	1.27	49.35

**Figure 7:**
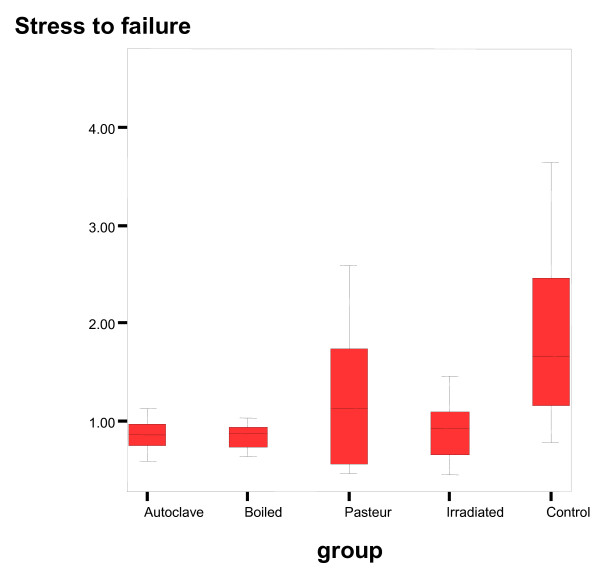
**shows the calculated material properties Stress to failure**. Material properties (mean, +/- standard error) of the bone samples, stress to failure for the four different treatment groups and the control group. Post-hoc Tukey tests found that autoclaving and boiling; to a less extent gamma irradiation significantly reduced the material properties (p < 0.05).

The ANOVA test done on the study groups and control is shown in table 2. Figure 7 shows 'stress to failure'. Post-hoc Tukey tests found that autoclaving and boiling, to a less extent irradiation significantly reduced the material properties (p < 0.05); see Table 2.

**Table 2:** Significance levels of between treatment material property differences from Tukey post-hoc tests.

	Autoclave	Boiled	Pasteurized	Irradiate
**Stress to failure**				
• Autoclave				
• Boiled	1.000			
• Pasteurization	0.880	0.834		
• Irradiation	0.998	0.993	0.972	
• Control	0.011*	0.008*	0.110	0.026*
				
**Modulus**				
• Autoclave				
• Boiled	0.961			
• Pasteurization	0.997	0.844		
• Irradiation	0.808	0.403	0.943	
• Control	0.014*	0.002*	0.035*	0.184

*p < (0.05)

Structural failure is initiated when the material is stressed to its strength limit, thus causing fracture or excessive deformations. The ultimate failure strength of the material is its maximum load-bearing capacity.

**Figure 8:**
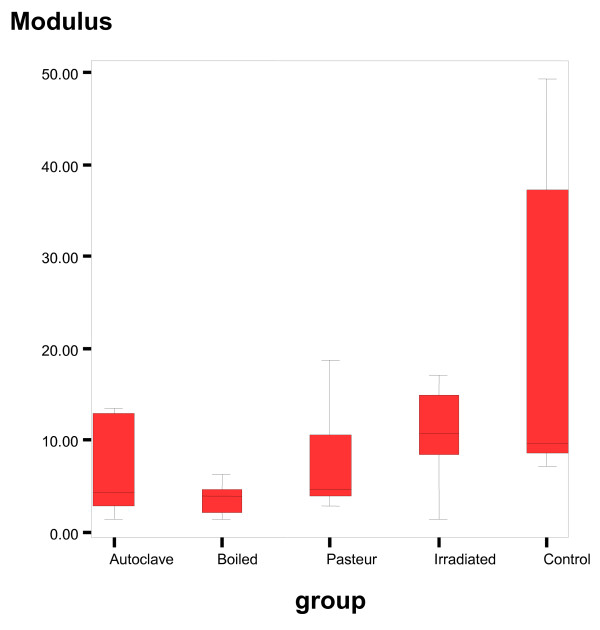
**Shows modulus to failure**. 'Modulus' for the 4 different treatment groups and the control group. Post-hoc Tukey tests found that autoclaving, boiling and pasteurization significantly reduced the material properties (p < 0.05).

Figure 8 shows 'modulus to failure'. Post-hoc Tukey tests found that autoclaving, boiling and pasteurization significantly reduced the material properties (p < 0.05); see Table 2.

The 'elastic modulus' was calculated by a linear regression over the steepest part of the stress*-*strain curve before any modulus reduction occurred. The 'Modulus of Elasticity' can be used to determine stress-strain relationships in the linear-elastic portion of the stress-strain curve. The linear-elastic region is taken to be between 0 and 0.2% strain, and is defined as the region of strain in which permanent deformation occurs.

Post-hoc Tukey HSD tests found that irradiation significantly reduced structural property (p < 0.05); see Table 3.

**Table 3:** This is the main ANOVA result. The significance value comparing the groups

		Sum of Squares	df	Mean Square	F	**Sig**.
**Stress to Failure (**MPa)	Between Groups	9.112	4	2.278	4.287	.005
	Within Groups	23.914	45	.531		
	Total	33.026	49			
**Strain To Failure **(%)	Between Groups	221.920	4	55.480	3.275	.019
	Within Groups	762.395	45	16.942		
	Total	984.315	49			
**Modulus **(MP)	Between Groups	1356.686	4	339.171	4.765	.003
	Within Groups	3203.147	45	71.181		
	Total	4559.832	49			

Deformation of the material is the change in geometry when stress is applied. Strain or reduced deformation is the deformation change among the material field. For uniaxial loading - displacements of a specimen (for example a bar element), it is expressed as the quotient of the displacement and the length of the specimen.

A Tukey HSD post-hoc analysis revealed that pasteurization was the only treatment that did not significantly affect any material or structural properties of the cortical bone samples (considering its effect on the modulus which is significant but comparatively less) when compared to the controls. The autoclave and boiled samples, however, showed much poorer performance than the other two forms treatment. The 'stress to failure' strength reductions were 55% and 57% (p value < 0.05) respectively. 'Modulus' was reduced by 66% and 75% (p < 0.05) respectively. With the exception, 'strain to failure' was insignificant (p value > 0.05). The properties of the irradiation treated bones were the only sample that showed significant reduction by 62% (p value < 0.05) in 'strain to failure' compared to the other treated bones, and 50% reduction of 'stress to failure' with the exception of 'modulus'(as shown in table 2) All significance levels of material and structural property between-group differences are shown in Tables 3 and 4 respectively.

**Table 4:** Significance levels of between-treatment structural property differences from Tukey post-hoc tests.

	Autoclave	Boiled	Pasteurized	Gamma irradiate
**Strain to failure**				
• Autoclave				
• Boiled	1.000			
• Pasteurization	0.969	0.972		
• Irradiation	0.981	0.979	0.761	
• Control	0.065	0.068	0.239	0.016*

*p < (0.05)

### Histopathology results

**Figure 9:**
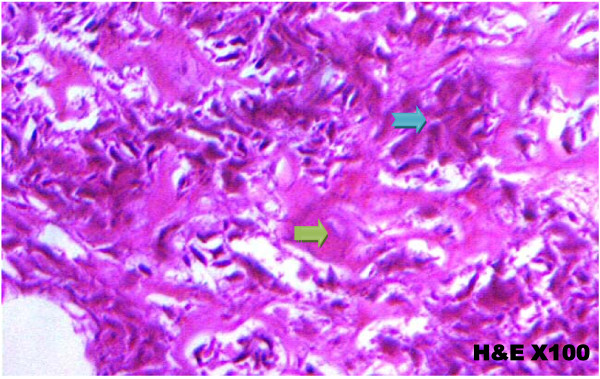
**Histology of Control group showing viable tumour cell with tumour osteoid Viable Tumour cells (Blue arrow); Tumour Osteoid (Green arrow)**.

**Figure 10:**
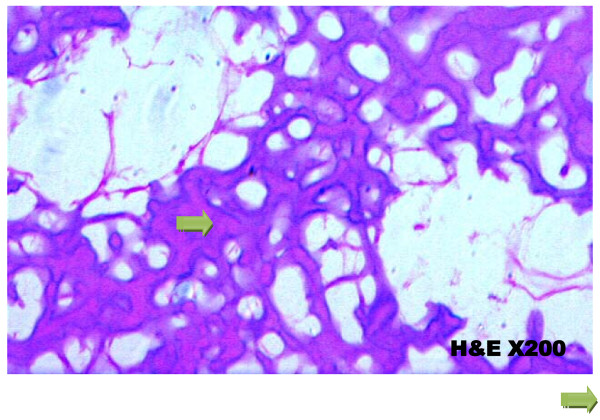
**Histology of sterilized specimen showing necrotic tumour osteoid( )**. No viable tumour cells.

Slides were prepared with hemotoxylin and eosin staining, read under light microscope. The histopathological results are shown in table 5. Figure 9, shows histology of the control group, figure 10 shows histology of the sterilized specimen with necrotic tumour osteoid. Sections of the bone specimens which have undergone treatment did not reveal any viable tumour cells. Sections of bone from the control group, which did not undergo any form of sterilization procedure, had viable tumor cells. The minimum heat treatment in this study is pasteurization (60°C for 30 minutes) and it was sufficient for complete sterilization of the tumour bone specimen. All the four methods of sterilization gave 100% tumour necrosis.

**Table 5:** Histopathological findings of the treated and untreated groups.

	A (Autoclave)	B (Boiled)	C (Control)	D (Pasteurized)	E (Irradiation)
Sample 1	LB/Os/NVT	LB/NVT	VT	LB/NVT	LB/NVT
Sample 2	Os/NVT	Os/NVT	VT	LB/Os/NVT	LB/NVT
Sample 3	LB/Os	Os/NVT	VT	Os/NVT	Os/NVT
Sample 4	LB/NVT	Os/NVT	VT	Os/NVT	LB/Os/NVT
Sample 5	LB/OsNVT	Os/NVT	Osteoid	LB/Os/NVT	Os/NVT
Sample 6	LB/NVT	LB/NVT	VT	LB/Os/NVT	Os/NVT
Sample 7	LB/Os/NVT	LB/NVT	VT	LB/NVT	LB/Os/NVT
Sample 8	Os/NVT	Inflammatory	VT	LB/Os/NVT	LB/Os/NVT
Sample 9	LB/Os/NVT	Os/NB/NVT	VT	Os/NVT	LB/Os/NVT
Sample 10	LB/Os/NVT	LB/NVT	VT	Os/NVT	LB/Os/NVT

Lamellar bone = LB

No Viable Tumour = NVT

Osteoid = Os

Viable tumour = VT

## Discussion

To produce effective treatment of bone tumours, it is essential to eradicate all tumour tissue. Extracorporeal devitalisation and reimplantation requires both a safe margin of resection and also definite eradication of all tumour cells. In 1928, Friedgood [6] was the first to show that Walker rat sarcoma cells were rendered non viable after heat treatment at 44°C for 30 minutes and many studies have since shown that the thermal doses for different tumour cell lines were on the same scale (Pincus and Fischer 1931 [7]; Johnson 1940 [8]; Selawry, Goldstein and McCormick 1957 [9]; Auersperg 1966 [10]; Rivard 1984 [11]; Inokuchi et al 1991 [12]). Certain devitalisation of tumour tissue requires as an absolute minimum two minutes at 60°C or 0.5 minutes at 65°C.

There have been numerous investigations utilizing pasteurization as a method of sterilization (Campanacci and Laus 1980 [13]). They conclude that autoclaving as a safe method for devitalising bone tumours (Enneking and Flynn 1969 [14]).

Kohler et al (1986) [15] demonstrated that there is rapid penetration of heat into the diaphyseal bones of rabbits during autoclaving at 121°C for 20 minutes and at 131°C for two minutes, respectively, and concluded that the method can be used safely for uniform and complete sterilisation of entire specimens.

The inductive capacity of bone is destroyed to a large extent after autoclaving (Burwell 1966 [16]; Urist and Hernandez 1974 [17]; Kreicbergs and Kohler 1989 [18]), and its osteoinductivity is also reduced with increasing temperature and increasing duration (Bernstein et al 1993 [19]). An increase of temperature between 80°C and 134°C is reported to reduce healing (Knaepler et at 1992 [20]). It is recommend to use minimum effective autoclaving time of 15 minutes at 134°C when heating large bone specimens to devitalise tumour cells.

This study is designed to examine the effects of autoclaving, boiling, pasteurization and gamma irradiation on the compressive strength and viability of the tumour cells in the recycle tumour bone.

In a bone biomechanical studies, bending, tension, compression, and torsion strength are usually measured. We studied the compression test as the transplanted bone is usually exposed to such loads in vivo.

There are several papers reporting the effect of heat treatment itself on the bone in regard to mechanical strength changes. Köhler et al [17] reported in a study using the diaphyseal bone of rabbits that the strength decreased to 77% in a torsional test after being autoclaved at 121°C for 20 min. Knaepler et al [21] reported in his study using pig cancellous bone that the compressive strength decreased to approximately 60% after 100°C treatment, but the mechanical strength was not influenced at 60°C of heat treatment.

Using data for compressive stress to failure, modulus and strain to failure, we could demonstrate there were significant effects of the various treatment procedures on these variables.

In our study after heat treatment at 100°C and above as in autoclave and boiled group, the stress to failure strength reduction was 55% and 57% respectively in the compression testing. While after heat treatment at 60°C (pasteurization), no significant decrease in strength was observed.

These results suggest that heat treatment over 100°C results in a decrease in the bone mechanical strength, but at 60°C, the heat treatment does not affect the mechanical strength of the bone.

Autoclaved and boiled sample revealed reduction in modulus by 66% and 75% (p value less than 0.05), respectively. With the exception to the strain to failure on which there were no significant effect (p value more than 0.05).

In contrast to the pasteurization and photon irradiation results, the data suggest that high temperatures significantly alter the compressive modulus and strength of the bone.

The photon irradiation treated bones were the only sample that showed significant reduction by 62% (p value less than 0.05) in strain to failure result compared to the other treated bones, and 50% reduction in stress to failure. There was no change in the modulus.

It is known that bone collagen attributes to the bone strength, and that its properties are changed by heating. Vangsness et al [22] reported that collagen structure changes at temperatures higher than 80°C. While Urist et al [23] reported that bone collagen did not shrink with temperatures below 60°C. These reports suggest that bone collagen degenerated at 100°C heat treatment, causing a decrease in the mechanical strength, while heat-treated bone at 60°C was not affected. Moreover, the decrease in the mechanical strength of heat-treated bone at 100°C was observed in the torsional test rather than the compression test. The bone strength against compression is mainly related to the bone density [24], while bone strength against torsioning is likely affected by the degeneration of collagen [25]. Therefore, apparent decrease was observed in our torsional strength testing.

The cause of this dramatic degradation in the compressive modulus and strength is most likely due to damage of the bone microstructure due to heating. Whatever the cause, these data show that heat treatments of allografts or autografts should be avoided. By autoclaving trabecular bone, the strength and stiffness of the material is reduced to half of its normal. Thus, it dramatically increases the chances of early graft failure in these cases.

Histopathology study of the sterilized specimens revealed that heat treatment at minimum of 60°C for 30 minutes is adequate to produce total tumour cell eradication.

Thermobiological research has shown that it is not necessary to heat to 100°C to cause tumour cells necrosis, but more research is needed to determine the minimum exposure time at lower temperatures which will guarantee devitalisation of tumour cells.

Hatano et al [26] conducted a histological study and confirmed that single radiation dose of 60 Gy was adequate for complete eradication of tumour cells in grafts.

Araki N et al, Davidson AW et al and Uyttendaele et al [27-29] reported their experience of more than 70 cases without local recurrence and metastatic disease suggests that the a single radiation dose of 50 Gy is sufficient. Higher doses have been shown to reduce the revascularization and osteoconductive capability of the graft, thereby increasing the time to union and incorporation [30-32]

Autoclaving excised bone tumours as a method of eradicating the neoplastic cells has been described but has the great disadvantage of causing a marked deterioration in the biological and biomechanical properties of the resected bone [33]

The effect of these methods of sterilization on its osteoconductive and osteoinductive properties of autologous tumour bone is not studied here but is currently being investigated in animal models. In our center, we are currently using pasteurization as a method of tumour sterilization.

## Conclusions

Sterilization of cortical bone sample by pasteurization and to a lesser extent, gamma irradiation does not significantly alter the mechanical properties when compared with untreated samples. All methods of sterilization give rise to total tumour kill. Mechanical properties of the bone degrade with the use of high temperature (greater than 100 degrees) such as boiling and irradiation.

## 7.1: Limitation of the study

The cortical bone samples for mechanical testing were resected just adjacent to the tumour mass. On macroscopic observation, the samples taken appeared uniform, therefore we assume that the tumour involvement of the bone is homogenous. But the fact that the specimen is taken just adjacent from the tumour mass, the microscopic quality of bone is likely to be altered. This may alter the mechanical testing of the specimen.

The sample size of ten in each group has an effect on the power of the analysis contributing to the study. A larger sample size would give better representation.

The study will be better supported with an in vivo study in animals on the osteoinductive properties of this sterilized bone when re-implanted back into the study subjects.

## Competing interests

The authors declare that they have no competing interests.

## Authors' contributions

VAS: Design and supervised the study, Main surgeon who operated on the cases and edited the final write up.

JN: Collected the samples and conducted the study. Wrote the draft for the paper.

MS: Provided the Radiotherapy for the specimens and the oncology input.

JP: Read all the histopathology slides and provided the input.

VAS planned and designed the study, carried out the surgery, harvest the specimens and edited the write up. JN carried out the test on the specimen, did the statistical anaylsis and wrote the draft of the study. MS provided radiotherapy services and advice for the study. JP did the histopathological part of the study. All authors read and approved the final manuscript.
